# SPIRITUAL RESILIENCE? THE ROLE OF RELIGIOSITY IN BUFFERING THE EFFECT OF NEIGHBORHOOD DISORDER ON COGNITIVE DECLINE

**DOI:** 10.1093/geroni/igac059.1995

**Published:** 2022-12-20

**Authors:** Haena Lee, Yeon Jin Choi, Jong Hyun Jung

**Affiliations:** USC, Los Angeles, California, United States; University of Southern California, Los Angeles, California, United States; Sungkyunkwan University, Seoul, Seoul-t'ukpyolsi, Republic of Korea

## Abstract

Living in neighborhoods with high levels of disorder and danger can induce psychological distress and compromise cognitive function. However, not all individuals who live in difficult life circumstances have poor health outcomes. Research on resilience shows that some older adults maintain healthy profiles despite adversity, but this has not been tested with respect to cognitive aging. In this paper, we focus on religiosity–religious belief and attendance–as a source of resilience and how it can reverse or reduce cognitive risks in later life that result from long-term exposure to neighborhood disorder. We used 2006-2016 Health and Retirement Study (HRS) to investigate how religiosity moderates the relationship between neighborhood disorder and cognitive decline. We assessed trajectories of cognitive functioning using the Telephone Inventory for Cognitive Status. We measured neighborhood disorder and neighborhood unsafety using the 2006/2008 HRS interviewer observation data and Housing data. We found that individuals living with higher levels of neighborhood disorder had lower cognitive functioning at baseline. The disorder effect was mitigated by religious belief—for instance, poor neighborhood conditions were negatively associated with cognitive function only for those with lower religious belief. The protective effect of religious belief was more pronounced among older Black women. This is consistent with prior literature that spirituality serves as a protective factor in the African American community, especially among women, for triumphing over adversity and lack of secular resources over the life course.

